# Almost half of references in reports on new and emerging nondrug health technologies are grey literature

**DOI:** 10.5195/jmla.2019.539

**Published:** 2019-01-01

**Authors:** Kelly Farrah, Monika Mierzwinski-Urban

**Affiliations:** Research Information Specialist, Canadian Agency for Drugs and Technologies in Health (CADTH), Ottawa, ON, Canada, kellyf@cadth.ca; Research Information Specialist, Canadian Agency for Drugs and Technologies in Health (CADTH), Ottawa, ON, Canada, monikam@cadth.ca

## Abstract

**Objective:**

The research investigated how frequently grey literature is used in reports on new and emerging nondrug health technologies, which sources are most cited, and how grey literature searching is reported.

**Methods:**

A retrospective review of references cited in horizon scanning reports on nondrug health technologies—including medical devices, laboratory tests, and procedures—was conducted. A quasi-random sample of up to three reports per agency was selected from a compilation of reports published in 2014 by international horizon scanning services and health organizations.

**Results:**

Twenty-two reports from 8 agencies were included in the analysis. On average, 47% (288/617) of references listed in the bibliographies of the horizon scanning reports were grey literature. The most frequently cited type of grey literature was information from manufacturers (30% of all grey literature references), regulatory agencies (10%), clinical trial registries (9%), and other horizon scans or evidence synthesis reports (9%). The US Food and Drug Administration (FDA) and ClincalTrials.gov were the most frequently cited specific sources, constituting 7% and 8% of grey literature references, respectively. Over two-thirds (15/22) of the analyzed reports provided some details on search methodology; all 15 of these reported searching some grey literature.

**Conclusions:**

In this sample, grey literature represented almost half of the references cited in reports on new and emerging nondrug health technologies. Of these grey literature references, almost half came from three sources: the manufacturers, ClincalTrials.gov, and the FDA. There was wide variation in the other sources cited. Literature search methodology was often insufficiently reported for analysis.

## INTRODUCTION

Information on the potential impact of new and emerging health technologies can assist health care professionals and decision-makers make evidence-based choices about the use and implementation of technologies before they diffuse into the health care system. Horizon scanning, sometimes called early alert and awareness, is “the systematic identification of new and emerging health technologies that have the potential to impact on health, health services, and/or society” [1]. Internationally, numerous services and health organizations conduct horizon scanning [1]. Many of these agencies produce reports summarizing the information available on technologies that are just entering the market or technologies a few years away from market approval. These reports generally include details such as the intended use of the technology, available clinical evidence, cost, current regulatory status, safety, and potential implementation issues [2].

Because new and emerging technologies have not yet diffused widely, literature about these technologies in traditional biomedical bibliographic databases, such as MEDLINE, is often more limited than literature about established health technologies. As such, grey literature is expected to play an important role in horizon scanning reports. The Grey Literature Network Service (GreyNet) defines grey literature as “a field in library and Information science that deals with the production, distribution, and access to multiple document types produced on all levels of government, academics, business, and organization in electronic and print formats not controlled by commercial publishing i.e. where publishing is not the primary activity of the producing body” [3]. For example, grey literature can include reports from governmental or regulatory agencies, information on manufacturer websites, unpublished trials, or conference presentations.

Scanning individual websites has long been recognized as an important way of identifying new and emerging health technologies. A 2003 survey of 10 agencies involved in horizon scanning found that the majority (7/10) systematically scanned an average of 11 to 27 websites per agency [4].

Identifying information on new and emerging nondrug health technologies—which include medical devices, laboratory tests, and surgical procedures—can be more challenging than for pharmaceuticals. These technologies are diverse in nature, encompassing devices such as breast implants, catheters, and surgical lasers as well as surgical techniques and genetic testing.

Compared with pharmaceuticals, nondrug health technologies have different development and regulatory pathways. Medical devices often follow a less linear development pathway [5], and, for lower-risk devices, approval is more rapid and less rigorous [6]. In the United States and elsewhere, many medical devices are approved for use before clinical studies on the new device are published [6]. These devices are approved through the Food and Drug Administration’s (FDA’s) 510(k) pathway, which allows accelerated regulatory approval for devices that are considered equivalent to another device that is already on the market [6]. The lack of published literature that is available for these technologies entering the market increases the importance of grey literature for decision-making on these new devices.

A 2016 survey of international horizon scanning services shows that most conduct grey literature searches in preparing their reports [7]; however, it is unclear how frequently grey literature is cited and, when it is cited, which types of grey literature and sources are most frequently used in completed reports on nondrug health technologies. Having this information could help make grey literature searching more standardized and efficient by identifying the most productive sources. This project investigated the use of grey literature in horizon scanning reports on nondrug health technologies published by international agencies, including how often it is cited, which sources are most frequently cited, and how grey literature searching is reported.

## METHODS

The authors conducted a retrospective review of horizon scanning reports on nondrug health technologies, including medical devices, laboratory tests, biomarkers, and procedures.

### Sample selection

*Horizon Scan Roundup – 2014* from the Canadian Agency for Drugs and Technologies in Health (CADTH) [8] was used to identify relevant reports to include in this study. At the time of study, the 2014 edition was the most recent one available. *Horizon Scan Roundup* is a compilation of 130 titles published in 2014 by major international horizon scanning services and health organizations. Items included in the *Horizon Scan Roundup* are restricted to nondrug health technologies (including medical devices, laboratory tests, biomarkers, programs, and procedures) that could have “a significant impact on health care in Canada” [8].

A quasi-random sample of reports from the *Horizon Scan Roundup* was selected. Reports with no references listed were excluded. Up to three reports from each agency were randomly selected using the online random number generator from RANDOM.ORG [9]. If an agency had three or fewer reports listed, all reports were included by default.

### Data collection

Reference lists of the selected reports were screened by a single reviewer. Each reference was categorized as grey literature or not grey literature according to the GreyNet definition of grey literature [3]. A second reviewer confirmed the classification. Disagreements were resolved by discussion between the two reviewers until consensus was reached. Citations of expert opinion (e.g., personal communications with a physician) listed in the report bibliographies were excluded from analysis.

The source of each grey literature reference was recorded. Additionally, each grey literature reference was classified by type using prespecified categories. For all reports, the percentage of grey literature references compared with non-grey literature references was calculated.

The reports were also screened to identify information on the literature search methods, including whether search methods were described, if grey literature was searched, and, if so, which sources were searched.

## RESULTS

### Included reports

Twenty-two reports from eight different agencies were included in the analysis. Table 1 provides information on the agencies and number of reports included.

**Table 1:** Agencies and number of reports included

Agency	Country	# of Reports
Agenzia Nazionale per i servizi sanitari Regionali (Agenas)*	Italy	1
Agency for Healthcare Research and Quality (AHRQ)	United States	3
Canadian Agency for Drugs and Technologies in Health (CADTH)	Canada	3
ECRI Institute	United States	3
Health Policy Advisory Committee on Technology (HealthPACT)	Australia	3
National Evidence-Based Healthcare Collaborating Agency (NECA) Horizon Scanning Service of Innovative Global Health Technology (H-SIGHT)	South Korea	3
National Institute for Health and Care Excellence (NICE)	United Kingdom	3
National Institute for Health Research (NIHR) Horizon Scanning Research & Intelligence Centre (HSRIC)	United Kingdom	3
Total		22

* Only one report from Agenas was listed in the *Horizon Scan Roundup.*

### Proportion of grey literature references

On average, 47% (288/617) of references listed in the bibliographies of the 22 horizon scanning reports reviewed were grey literature. The median percentage of grey literature references cited in individual reports was 42%, with a range of 0 (0/4 references) to 87% (20/23 references).

### Type of grey literature references

The most frequently cited type of grey literature was documents produced by manufacturers of the technologies under review (30% of all grey literature references, including press releases found on manufacturer websites). Table 2 details the number of grey literature results by type.

**Table 2:** Number of grey literature references by type

Grey literature type	References	

	n	(%)
Manufacturer information*	87	(30%)
Regulatory agency	28	(10%)
Clinical trial registry	26	(9%)
Horizon scan/rapid review/health technology assessment	25	(9%)
Health plan policy	21	(7%)
News release†	18	(6%)
Statistics	17	(6%)
Background‡	17	(6%)
Guideline	11	(4%)
Economic information	9	(3%)
Unindexed online journal/magazine	9	(3%)
Conference abstract	8	(3%)
Other (e.g., presentation, hearing, form)	7	(2%)
Safety advisory	5	(2%)
Total	288	(100%)

* Includes press releases posted on manufacturer websites.

† Excludes press releases posted on manufacturer websites.

‡ Includes general information on websites of societies and associations, government factsheets, etc.

### Sources of grey literature

Information from ClincalTrials.gov (8%, n=24) and the FDA (7%, n=21) were the most frequently cited specific sources of grey literature. There was great diversity among all other sources cited in the horizon scanning reports, each appearing with a frequency of less than 5% in the bibliographies of all reports.

### Search methodology reported

Out of the 22 reports reviewed, 9 (41%) provided the full literature search methodology for the report, 6 (27%) provided only partial search methodology (i.e., reported methods for one specific section of the report only), and 7 (32%) did not provide any details on the literature search. All 15 reports that provided full or partial details on the search methodology described searching some grey literature sources.

Table 3 displays grey literature sources listed as searched in the reports’ methods sections. Of the fifteen reports that described searching grey literature, nine reported searching ClincalTrials.gov. Eleven of the fifteen reports reported searching the Cochrane Library, which contains several databases including the Health Technology Assessment Database, which indexes grey literature. However, it was unclear from the methodology of most reports which Cochrane Library databases were searched. Other sources mentioned were only searched by one agency or only listed in one report.

**Table 3:** Grey literature sources listed in search methods

Source	# of Reports
Cochrane Library*	11
ClincalTrials.gov	9
Australian New Zealand Clinical Trials Registry	3
CADTH Grey Matters	3
US Food and Drug Administration (FDA)	1
Medicines & Healthcare products Regulatory Agency (MHRA)	1
Tinnitus Research Initiative conference abstracts	1

* The Cochrane Library contains several databases, including the Health Technology Assessment Database, which indexes grey literature.

It was unclear from the methodology of most reports which Cochrane Library databases were searched.

## DISCUSSION

Grey literature represented a large proportion of references cited in this study of horizon scanning reports on nondrug health technologies: almost half of references (47%) were classified as grey literature. This proportion of grey literature is higher than that found in a similar study on rapid reviews, which reported that 23% of references listed were grey literature [10]. The higher percentage of grey literature in horizon scanning reports reflects a greater reliance on this type of information for new and emerging health technologies.

Of the grey literature references, manufacturers were a key source of information, with a third of grey literature references originating from the manufacturers of the technologies being reported about. It was not surprising that the manufacturers were a key source of information given the technologies’ early stage of development and limited diffusion.

Although the 2003 survey by Douw et al. [4] assessed how horizon scanning agencies used websites to identify new health technologies rather than the type of information cited in reports, it was interesting to compare their results with those of the present study. While the grey literature landscape has changed greatly since 2003, given developments in Internet searching and website growth in the past decade, there are similarities in the types of sources that were searched. In both studies, regulatory agency sites and press releases were often used. However, new types of sources that have been developed in the intervening years, such as clinical trial registries, are now increasingly important. Similar to our results, Douw et al. found wide variation in the specific sites searched: 110 different sites across 6 agencies, with little overlap in the level of importance the agencies ranked to each individual site. The lack of commonality in sites searched and cited might result from differences in the agencies’ scope, including the types of technologies assessed [4].

At the outset of this research, we had hoped to create a list of key sites to search for grey literature on new and emerging nondrug technologies, similar in concept to CADTH’s *Grey Matters* checklist [11]. We found that almost half of the grey literature cited came from three sources: manufacturers of nondrug health technologies, ClincalTrials.gov, and the FDA. There was great variation in the sources of the remaining references. Due to heterogeneity in the other grey literature sources that were cited, our results implied that it would be difficult to create one standard checklist of key websites for identifying grey literature across all types of nondrug technologies. Additionally, it was difficult to determine which grey literature sources were searched for many reports because, in over half of the analyzed reports, the methodology used for grey literature searching was only partially stated or not reported at all.

A limitation of this research was that our sample included only twenty-two reports on nondrug health technologies that were available in the English language and were gathered from a single source. While these reports reflected a variety of countries, organizations, and technology types, the small sample and language restriction might limit the generalizability of the results. Further, this study only examined whether references included in the report were grey literature and did not examine how the content of the grey literature cited was utilized in the reports. We also could not determine whether grey literature sources were consulted by report authors and found helpful but were not specifically cited. Further research is needed to examine the context in which grey literature is used in horizon scanning reports.

This research provides evidence of the value of grey literature as a source of information on new and emerging nondrug health technologies, especially information from regulatory agencies such as the FDA and clinical trial registries such as ClincalTrials.gov. The results also have implications for information specialists and others who perform searches that support horizon scanning activities. In particular, it emphasizes the need to search sources beyond standard biomedical bibliographic databases, such as MEDLINE and Embase, to identify a large portion of information on these technologies. Given the heterogeneity of sources outside the top three cited, however, having a single checklist of grey literature sources to guide searching may be impractical. Instead, information specialists and other searchers seeking information on new and emerging nondrug technologies could consider focusing their efforts on search engines and aggregator sites. Further, we found that literature search methods were often insufficiently reported or not reported at all. Improving reporting of literature searching to include details on whether grey literature was searched and which sources were searched would enhance the transparency and reproducibility of horizon scanning reports.
